# Robust Association Between Changes in Coronary Flow Capacity Following Percutaneous Coronary Intervention and Vessel-Oriented Outcomes and the Implication for Clinical Practice

**DOI:** 10.3389/fcvm.2022.901941

**Published:** 2022-06-15

**Authors:** Rikuta Hamaya, Taishi Yonetsu, Kodai Sayama, Kazuki Matsuda, Hiroki Ueno, Tatsuhiro Nagamine, Toru Misawa, Masahiro Hada, Masahiro Hoshino, Tomoyo Sugiyama, Tetsuo Sasano, Tsunekazu Kakuta

**Affiliations:** ^1^Division of Preventive Medicine, Department of Medicine, Brigham and Women’s Hospital and Harvard Medical School, Boston, MA, United States; ^2^Department of Epidemiology, Harvard T.H. Chan School of Public Health, Boston, MA, United States; ^3^Department of Cardiology, Tokyo Medical and Dental University, Tokyo, Japan; ^4^Department of Cardiology, Tsuchiura Kyodo General Hospital, Tsuchiura, Japan

**Keywords:** coronary flow capacity, coronary flow reserve, percutaneous coronary intervention, fractional flow reserve, coronary artery disease

## Abstract

**Background:**

Coronary flow capacity (CFC) is a potentially important physiologic marker of ischemia for guiding percutaneous coronary intervention (PCI) indication, while the changes through PCI have not been investigated.

**Objectives:**

To assess the determinants and prognostic implication of delta CFC, defined as the change in the CFC status following PCI.

**Materials and Methods:**

From a single-center registry, a total of 450 patients with chronic coronary syndrome (CCS) who underwent fractional flow reserve (FFR)-guided PCI with pre-/post-PCI invasive coronary physiological assessments were included. Associations between PCI-related changes in thermodilution method-derived CFC categories and incident target vessel failure (TVF) were assessed.

**Results:**

The mean (*SD*) age was 67.1 (10.0) years and there were 75 (16.7%) women. Compared with patients showing no change in CFC categories after PCI, patients with category worsened, +1, +2, and +3 category improved had the hazard ratio (95% *CI*) for incident TVF of 2.27 (0.95, 5.43), 0.85 (0.33, 2.22), 0.45 (0.12, 1.63), and 0.14 (0.016, 1.30), respectively (*p* for linear trends = 0.0051). After adjustment for confounders, one additional change in CFC status was associated with 0.61 (0.45, 0.83) times the hazard of TVF. CFC changes were largely predicted by the pre-PCI CFC status.

**Conclusion:**

Coronary flow capacity changes following PCI, which was largely determined by the pre-PCI CFC status, were associated with the lower risk of incident TVF in patients with CCS who underwent PCI. The CFC changes provide a mechanistic explanation on potential favorable effect of PCI on reducing vessel-oriented outcome in lesions with reduced CFC and low FFR.

## Introduction

Globally, clinical practice is getting toward choosing a deferral of percutaneous coronary intervention (PCI) in patients with chronic coronary syndrome (CCS) given comparative effectiveness of PCI against medical therapy (1–4) with respect to patient outcomes. PCI is a costly procedure with potential adverse effects (5), and hence the patient selection for the intervention should be very strict especially intending to reduce future adverse events. On the contrary, deferring all elective PCIs in patients with CCS might be too simplistic, given evidence showing the effects of fractional flow reserve (FFR)-guided PCI on reducing spontaneous myocardial infarction or future revascularization (6, 7), and the prognostic benefit of PCI differential according to several factors (1, 2, 8). Recently, PCI is principally guided by FFR or instantaneous wave-free ratio (iFR), whereas integrating complementary characteristics for the purpose is emergingly warranted to tailor the intervention and maximize the clinical benefit.

Coronary flow capacity (CFC) is a relatively new, theoretically grounded physiological index that represents ischemia due to coronary flow limitation (9–12). Reduced CFC is a condition with low coronary flow reserve [CFR; hyperemic coronary flow (hCF) divided by resting CF] combined with slow hyperemic CF rather than fast resting CF. CFC holds interesting prognostic information where severely reduced CFC does not necessarily implicate elevated risk for future cardiovascular events if treated by PCI, whereas low CFR does regardless of PCI treatment (12, 13). Thus, reduced CFC may highlight a reversible feature of ischemic burden through revascularization, and we have previously reported the potential utility of CFC in guiding PCI to improve the overall prognostic benefit (12, 13). However, the change in CFC status following PCI, which is an important measure of assessing the impact of PCI with respect to the flow restoration and the consequent impact on clinical courses, has not been investigated.

In the present study, to fill the knowledge gap, we aimed to assess the prognostic implication of delta CFC, defined as the changes of CFC status following PCI. We also evaluated the predictability of delta CFC. We hypothesized that delta CFC would be associated with vessel-related outcomes and it would be predominantly determined by the pre-PCI CFC status.

## Materials and Methods

### Population

From January 2011 to April 2019, patients with known CCS who underwent PCI with the measurements of both pre- and post-PCI comprehensive coronary physiological assessments at Tsuchiura Kyodo General Hospital were identified from the institutional database. We excluded patients with indications for revascularization of ≥2 vessels, angiographically significant left main disease, previous CABG, renal insufficiency with baseline creatinine > 2.0 mg/dl, decompensated heart failure, cardiogenic shock, acute myocardial infarction, atrial fibrillation, extremely tortuous, or calcified coronary arteries, vessels with visible collateral development or ostial stenosis, and unreliable physiological assessment including CFR < 0.5 or CFR > 7. We did not exclude on the basis of the extent of stenosis outside of the above criteria, while subtotal lesions in which invasive physiological assessment could not be conducted were not included. The institutional ethics committee approved the study protocol. All patients provided written informed consent for enrollment in the institutional database for potential future investigations. All patient data and procedural details were obtained from medical records. The study complies with the Declaration of Helsinki.

### Percutaneous Coronary Intervention and Multivessel Disease

Percutaneous coronary intervention was indicated according to clinical practice guidelines at the time of the procedure with necessarily presence of ischemia evaluated by FFR, stress echocardiogram, cardiac magnetic resonance tomography, coronary computed tomography, single-photon emission computerized tomography, or the combinations, and agreement between ≥2 board-certified cardiologists. The diseased vessel was defined as main branches having ≥50% stenosis on visual assessment, and multi-vessel disease corresponded to coronary arteries with ≥2 angiographical diseased vessels.

### Coronary Physiological Assessment

Coronary physiological assessment was performed using thermodilution methods by using PressureWire (Abbott Vascular, St Paul, MN, United States) before and after PCI. After intracoronary nitrate (100 or 200 μg) administration, resting and hyperemic thermodilution curves were obtained in triplicate using three injections (3–4 ml each) of room-temperature saline, and the inverse of the average basal (bTmn) and hyperemic mean transit times (hTmn) were calculated. Hyperemia was induced by intravenous infusion of adenosine 5′-triphosphate (140–160 mg/kg/min). In vessels with tandem lesions, we optimized the treatment strategy in a standard way and conducted physiological assessment as follows: place the wire at the most distal of a target vessel to assess FFR, treat lesions where greater FFR step-up was observed, assess post-PCI physiological indices, and add PCI to residual treatable lesions with apparent FFR step-up (and if so again assess post-PCI physiological indices).

Fractional flow reserve was calculated as the ratio of mean distal coronary pressure (Pd) to mean aortic pressure (Pa) during maximal hyperemia. Basal (bCF) and hyperemic coronary flow (hCF) were defined as the inverse of bTmn and hTmn, respectively (14). CFR was calculated as the ratio of hyperemic to basal coronary flow. IMR was defined as hyperemic Pd * hTmn or hyperemic Pa * hTmn * [(1.35 * ratio of mean distal-to-aortic coronary pressure)–0.32] as detailed in elsewhere (15).

### Definition of Coronary Flow Capacity

**FIGURE 1:**
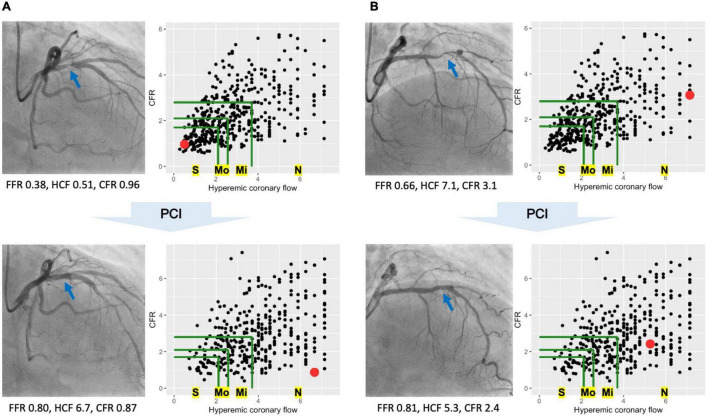
Coronary flow capacity (CFC) changes in two representative cases. Two representative cases showing distinct CFC changes following percutaneous coronary intervention (PCI). In each cine image, blue arrows indicate the culprit lesions in the left anterior descending arteries. Each scatter plot shows CFC map, where each dot representing one vessel is mapped according to the hyperemic coronary flow (hCF, x-axis) and coronary flow reserve (CFR, y-axis). Green lines are the boundaries of CFC categories; the bounded most inner to outer areas are corresponded to severely reduced (S), moderately reduced (Mo), mildly reduced (Mi), and normal CFC (N), respectively. Red dots in the CFC maps represent the cases of each cine image. **(A)** PCI increased the hCF from 0.51 to 6.7 with a little effect on CFR, leading to the improvement in CFC categories from severely reduced to normal ones. Benefit of PCI would be expected in such cases with greater CFC improvement (i.e., lower risk of target-vessel failure). **(B)** PCI did not let changes in CFC categories; pre-PCI normal to post-PCI normal CFC. In such cases with no CFC improvement following PCI, the improvement in fractional flow reserve (FFR) might indicate the modification in the epicardial lesions but not the coronary flow restoration, potentially highlighting the limited benefit of PCI.

Coronary flow capacity is a concept incorporating decreased CFR and reduced hyperemic coronary flow originally proposed in PET (9). Most previous studies characterized CFC status as severely reduced, moderately reduced, mildly reduced, and normal, linking them to definite, potential, unlikely, and no ischemia, respectively (10, 12, 16, 17). We defined the CFC status in line with previously published largest study using thermodilution technique (12); normal CFC as CFR ≥ 2.80 with hCF ≥ 3.70; mildly reduced CFC as CFR < 2.80 and ≥2.10, combined with hCF < 3.70 and ≥2.56; moderately reduced CFC as CFR < 2.10 and ≥1.70, and 1/Tmn < 2.56 and ≤2.00; and severely reduced CFC otherwise (CFR < 1.70 and hCF < 2.00). The same criteria were applied for the pre- and post-PCI physiological assessments. Figure 1 illustrates the changes of CFC status before/after PCI in two representative cases.

### Delta Coronary Flow Capacity

We ranked CFC categories as (1) for severely reduced, (2) for moderately reduced, (3) for mildly reduced, and (4) for normal. Delta CFC was defined as a numeric difference between post-PCI CFC minus pre-PCI CFC rank, ranging from -3 to +3; for example, +3 reflects the changes from severely reduced to normal CFC following PCI.

### Clinical Follow-Up

Patients were followed-up by outpatient clinic visits or by telephone contact to ascertain the occurrence of target vessel failure (TVF), defined as a composite of cardiac death, acute MI not clearly attributable to a non-target vessel (target-vessel MI; TVMI), and clinically driven revascularization of the target (PCI-treated) vessel (target-vessel revascularization; TVR). All patient-reported adverse events were verified by evaluating hospital records or contacting the treating cardiologist or general practitioner. All events were checked at least twice by different experienced cardiologists.

### Statistical Analysis

Continuous variables are presented as mean (*SD*) or median (Q1, Q3) and categorical variables are presented as counts (percentages). Missing values in covariates were imputed by classification and regression tree methods. Baseline characteristics according to the pre-PCI CFC status or CFC changes following PCI were compared based on the standardized mean differences (SMD).

The predictability of CFC changes was assessed for each pre-PCI characteristics, respectively, with use of area under the curve (*AUC*), sensitivity and specificity at the best cutoffs, and receiver operating characteristic (*ROC*) curves. The prediction for the continuous delta CFC was evaluated with the use of *R*-squared values.

Hazard ratios (*HRs*) of incident TVF were estimated by the COX proportional hazard models, either for categorical CFC changes [worsened, no change (reference), +1 to +3 categories improved] or of continuous delta CFC (per one category change). The *p*-values for linear trends were calculated from the COX models for continuous delta CFC. Models were adjusted for age (continuous), sex (men/women), diabetes (yes/no), vessel location (left anterior descending/left circumflex/right coronary artery), multivessel disease (yes/no), and FFR (continuous). Associations between pre- or post-PCI CFC categories and incident TVF were also assessed similarly. Relevant Kaplan–Meier curves were also computed.

The discrimination ability of incident TVF was assessed by various nested logistic regression models; Model 1 included age, sex, diabetes, vessel location, and multivessel disease; Model 2 was Model 1 plus pre-PCI FFR; Model 3 was Model 2 plus pre-PCI CFR (continuous); and Model 4 was Model 3 plus delta CFC (in ranks, ranging from -3 to +3). The improvements in the discrimination were assessed by net reclassification improvement (NRI) and integrated discrimination improvement (IDI).

The *p-*value for linear trend was calculated to estimate the statistical significance of the association of CFC in ranks (ranging from 1 to 4) or delta CFC (ranging from -3 to +3) and incident TVF in the COX proportional hazard models. Two-sided *p* values for linear trends < 0.05 were considered statistically significant. All analyses were conducted using R 4.0.3 (The R Foundation).

## Results

### Baseline Characteristics

A total of 450 patients with a clinical indication for revascularization and with 450 vessels with one coronary lesion indicated for and amenable to revascularization (1 vessel/subject) were included in the present analysis, and the mean (*SD*) age was 67.1 (10.0) years and there were 75 (16.7%) women. Median (Q1, Q3) FFR and CFR were 0.70 (0.63, 0.75) and 2.00 (1.33, 2.95), respectively. A total of 99, 63, 96, and 192 patients were classified as having severely reduced, moderately reduced, mildly reduced, and normal CFC status at baseline.

Table 1 summarizes the patient characteristics according to pre-PCI CFC status. The worse CFC status was associated with generally worse coronary physiologic profile. Medians (*IQRs*) FFR, CFR, and IMR were 0.62 (0.54, 0.69), 1.17 (1.03, 1.36), and 40.6 (35.0, 55.6) in patients with severely reduced CFC, and 0.73 (0.69, 0.78), 3.05 (2.28, 3.81), and 14.9 (11.0, 19.8) in those with normal CFC, respectively.

**TABLE 1:** Baseline characteristics by pre-revascularization CFC status.

Pre-PCI CFC	Severely reduced CFC	Moderately reduced CFC	Mildly reduced CFC	Normal CFC	SMD
	*N* = 99	*N* = 63	*N* = 96	*N* = 192	
Age, year	69.3 (10.7)	68.9 (10.6)	68.4 (9.3)	64.9 (9.3)	0.24
Female	25 (25.3)	8 (12.7)	14 (14.6)	28 (14.6)	0.16
Smoking					0.15
Never	72 (72.7)	52 (82.5)	77 (80.2)	143 (74.5)	
Past	25 (25.3)	10 (15.9)	17 (17.7)	45 (23.4)	
Current	2 (2.0)	1 (1.6)	2 (2.1)	4 (2.1)	
Hypertension	67 (67.7)	48 (76.2)	74 (77.1)	128 (66.7)	0.15
Diabetes	44 (44.4)	29 (46.0)	34 (35.4)	74 (38.5)	0.13
Hypercholestrolemia	53 (53.5)	36 (57.1)	55 (57.3)	138 (71.9)	0.23
eGFR, mL/min/1.73 m^2^	60 (24)	63 (24)	63 (22)	67 (22)	0.16
Left ventricular EF ≤ 50%	16 (16.2)	7 (11.1)	20 (20.8)	23 (12.0)	0.15
Multivessel disease	38 (38.4)	18 (28.6)	33 (34.4)	49 (25.5)	0.16
Vessel location					0.25
Right coronary artery	23 (23.2)	17 (27.0)	19 (19.8)	29 (15.1)	
Left anterior descending artery	67 (67.7)	34 (54.0)	65 (67.7)	138 (71.9)	
Left circumflex artery	9 (9.1)	12 (19.0)	12 (12.5)	25 (13.0)	
FFR, unit	0.61 [0.53, 0.69]	0.68 [0.60, 0.74]	0.72 [0.66, 0.75]	0.73 [0.69, 0.77]	0.78
CFR, unit	1.12 [0.96, 1.33]	1.76 [1.28, 1.90]	2.18 [1.64, 2.39]	3.12 [2.42, 3.94]	1.67
IMR, unit	41.0 [34.9, 56.1]	27.4 [23.4, 39.7]	20.9 [17.8, 29.8]	15.0 [11.0, 20.5]	0.96
Baseline coronary flow, unit	1.03 [0.74, 1.27]	1.14 [0.77, 1.70]	1.34 [0.98, 1.83]	1.28 [0.87, 2.17]	0.43
Hyperemic coronary flow, unit	1.15 [0.83, 1.49]	2.04 [1.44, 2.27]	2.78 [2.13, 3.12]	4.08 [3.03, 5.56]	1.81

*Values are n (%) for categorical variables and mean (SD) or median (IQR) for continuous variables.*

*CFC, coronary flow capacity; CFR, coronary flow reserve; EF, ejection fraction; FFR, fractional flow reserve; IMR, index of microvascular resistance; PCI, percutaneous coronary intervention; SMD, standardized mean difference.*

### Coronary Flow Capacity Changes Following Percutaneous Coronary Intervention

**FIGURE 2:**
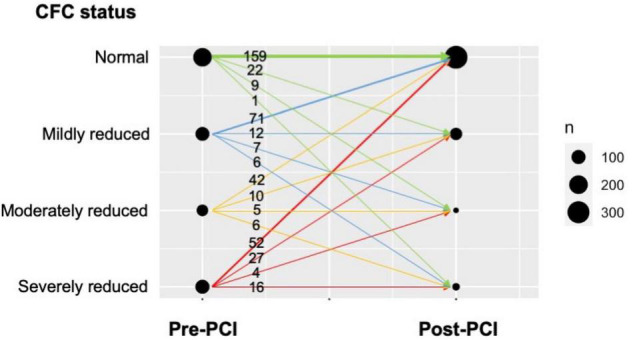
Changes in CFC following PCI in the present cohort. The plot visualizes the number of patients with respect to pre-PCI CFC, post-PCI CFC, and the change. Numbers indicate the number of patients corresponding to the CFC changes, illustrated as the arrows. Thickness of the arrows and circles reflect the number of patients in each CFC change and each pre-/post-CFC categories, respectively. The majority of the patients improved their CFC status into normal after PCI, while the proportion was smaller in those with pre-PCI reduced CFC.

Figure 2 illustrates the changes in CFC categories following PCI. In every pre-PCI CFC status, the majority were improved into normal CFC after PCI, leading to a total of 324 (80%) patients having post-PCI normal CFC status. Worse pre-PCI CFC status was associated with a higher probability of having worse post-PCI CFC status; for example, post-PCI moderately or severely reduced CFC was observed in 20 (24%), 11 (19%), 13 (14%), and 10 (6%) patients in pre-PCI severely reduced, moderately reduced, mildly reduced, and normal CFC status, respectively.

Characteristics of each delta CFC category are summarized in Table 2. Worsening, no change, +1, +2, and +3 rank changes in CFC categories following PCI were observed in *N* = 52, 192, 85, 69, and 52 patients, respectively. There were no clear trends in demographics across the groups, while lower FFR, CFR and hyperemic coronary flow, higher IMR, and worse CFC profiles were associated with greater CFC improvement. Those with worsened CFC after PCI were characterized with a relatively higher proportion of LAD lesions, while no other clear differences were found comparing with the patients with no changes in CFC.

**TABLE 2:** Characteristics by categories of CFC changes following PCI.

CFC change	Worsened	No change	+1 category improved	+2 category improved	+3 category improved	SMD
	*N* = 52	*N* = 192	*N* = 85	*N* = 69	*N* = 52	
Age, year	67.8 (9.4)	65.9 (9.6)	67.9 (9.5)	68.7 (10.3)	67.9 (12.1)	0.11
Female	9 (17.3)	30 (15.6)	13 (15.3)	8 (11.6)	15 (28.8)	0.19
Smoking						0.16
Never	41 (78.8)	147 (76.6)	67 (78.8)	52 (75.4)	37 (71.2)	
Past	11 (21.2)	41 (21.4)	16 (18.8)	15 (21.7)	14 (26.9)	
Current	0 (0.0)	4 (2.1)	2 (2.4)	2 (2.9)	1 (1.9)	
Hypertension	34 (65.4)	136 (70.8)	62 (72.9)	47 (68.1)	38 (73.1)	0.088
Diabetes	27 (51.9)	80 (41.7)	26 (30.6)	29 (42.0)	19 (36.5)	0.20
Hypercholestrolemia	37 (71.2)	130 (67.7)	50 (58.8)	35 (50.7)	30 (57.7)	0.26
eGFR, mL/min/1.73 m^2^	64 (24)	65 (23)	63 (25)	63 (23)	63 (21)	0.045
Left ventricular EF ≤ 50%	6 (11.5)	26 (13.5)	17 (20.0)	12 (17.4)	5 (9.6)	0.15
Multivessel disease	12 (23.1)	53 (27.6)	34 (40.0)	18 (26.1)	21 (40.4)	0.21
Vessel location						0.22
Right coronary artery	7 (13.5)	35 (18.2)	18 (21.2)	17 (24.6)	11 (21.2)	
Left anterior descending artery	42 (80.8)	130 (67.7)	56 (65.9)	41 (59.4)	35 (67.3)	
Left circumflex artery	3 (5.8)	27 (14.1)	11 (12.9)	11 (15.9)	6 (11.5)	
FFR, unit	0.72 [0.68, 0.77]	0.72 [0.69, 0.77]	0.72 [0.65, 0.75]	0.66 [0.59, 0.72]	0.56 [0.47, 0.63]	0.84
CFR, unit	2.52 [1.84, 3.17]	2.90 [1.93, 3.76]	2.12 [1.63, 2.38]	1.36 [1.09, 1.81]	1.17 [0.96, 1.37]	1.21
IMR, unit	19.1 [14.4, 26.6]	15.8 [11.2, 24.7]	21.8 [18.9, 31.5]	34.6 [25.1, 44.9]	38.3 [34.0, 51.6]	0.72
Baseline coronary flow, unit	1.27 [0.87, 2.11]	1.23 [0.87, 2.01]	1.30 [0.99, 1.69]	1.12 [0.72, 1.49]	0.99 [0.75, 1.22]	0.39
Hyperemic coronary flow, unit	2.90 [2.31, 4.09]	3.85 [2.56, 5.26]	2.70 [2.08, 3.12]	1.52 [1.12, 2.04]	1.07 [0.83, 1.50]	1.32
Pre-PCI CFC (%)						3.19
Severely reduced	33 (63.5)	159 (82.8)	0 (0.0)	0 (0.0)	0 (0.0)	
Moderately reduced	13 (25.0)	12 (6.2)	71 (83.5)	0 (0.0)	0 (0.0)	
Mildly reduced	6 (11.5)	5 (2.6)	10 (11.8)	42 (60.9)	0 (0.0)	
Normal	0 (0.0)	16 (8.3)	4 (4.7)	27 (39.1)	52 (100.0)	

*Values are n (%) for categorical variables and mean (SD) or median (IQR) for continuous variables.*

*CFC, coronary flow capacity; CFR, coronary flow reserve; EF, ejection fraction; FFR, fractional flow reserve; IMR, index of microvascular resistance; PCI, percutaneous coronary intervention; SMD, standardized mean difference.*

### Prediction of Coronary Flow Capacity Changes

**FIGURE 3:**
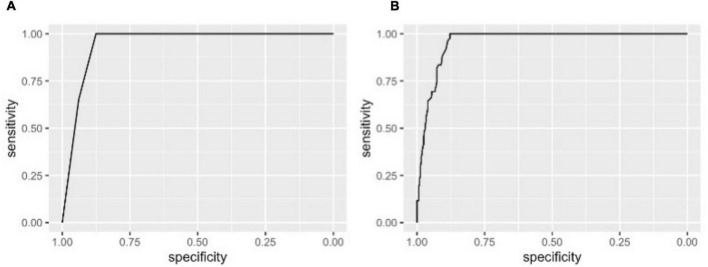
Receiver operating characteristic (ROC) curves for improvement in CFC by ≥2 categories following PCI. The ROC curves showing the discrimination of CFC improvement by ≥2 categories by pre-PCI CFC alone **(A)** and CFC plus FFR **(B)**. Area under the curves (*AUCs*) (95% *CI*) were 0.95 (0.93, 0.97) and 0.96 (0.94, 0.98) in panels **(A,B)**, respectively. Note, at the best cutoffs, the specificity was 100% because such improvement could only be observed in patients with pre-PCI severely or moderately reduced CFC.

The pre-PCI CFC status, although it has only 4 categories, was highly predictive of the improvement in CFC status following PCI, with AUC (95% *CI*) of 0.95 (0.93, 0.97) for ≥2 categories improvement (Figure 3A). The sensitivity was 100% because such improvement can only be possible in vessels with pre-PCI moderately or severely reduced CFC. Additional consideration of FFR had little influence on the discrimination (*AUC* [95% *CI*]: 0.96 [0.94, 0.98], Figure 3B). Other non-physiological characteristics were not comparatively predictive (Table 3A). Notedly, 48.6% of the variability of continuous delta CFC was explained solely by pre-PCI CFC, while only 12.4% by FFR (Table 3B). Results on the predictions for ≥1 and ≥3 CFC categories improvement were summarized in Tables 3C,D, which is consistently supporting the critical role of pre-PCI CFC in the predictions.

**TABLE 3:** Prediction of CFC improvement by various pre-PCI information.

	A. CFC improvement with ≥2 categories			
	Variable	AUC (95% CI)	Sensitivity	Specificity
Age	Continuous	0.55 (0.49, 0.61)	0.31	0.82
Sex	Discrete	0.52 (0.48, 0.56)	0.19	0.84
Smoking	3 categories	0.52 (0.47, 0.57)	0.26	0.78
Hypertension	Discrete	0.50 (0.45, 0.55)	0.30	0.71
Diabetes	Discrete	0.50 (0.45, 0.55)	0.60	0.40
Hypercholestrolemia	Discrete	0.56 (0.51, 0.61)	0.46	0.66
eGFR	Continuous	0.49 (0.43, 0.55)	0.49	0.57
Left ventricular EF ≤ 50%	Discrete	0.50 (0.47, 0.54)	0.86	0.15
Multivessel disease	Discrete	0.51 (0.46, 0.56)	0.32	0.70
FFR	Continuous	0.77 (0.72, 0.82)	0.72	0.73
CFR	Continuous	0.87 (0.84, 0.90)	0.96	0.69
IMR	Continuous	0.83 (0.80, 0.87)	0.89	0.68
Baseline coronary flow	Continuous	0.62 (0.57, 0.68)	0.78	0.45
Hyperemic coronary flow	Continuous	0.90 (0.87, 0.93)	0.98	0.74
Pre-PCI CFC	4 categories	0.95 (0.93, 0.97)	1.00	0.88

**B. Continuous delta CFC (changes in category ranks)**				

				***R*-squared**

Age				0.008
Sex				0.00
Smoking				0.001
Hypertension				0.00
Diabetes				0.006
Hypercholestrolemia				0.015
eGFR				0.001
LVEF ≤ 50%				0.002
Multivessel disease				0.009
FFR				0.12
CFR				0.28
IMR				0.11
Baseline coronary flow				0.041
Hyperemic coronary flow				0.30
Pre-PCI CFC				0.49

**C. ≥3 CFC categories improvement (i.e., severely reduced to normal CFC)**				

	**AUC (95% CI)**	**Sensitivity**	**Specificity**	

Age	0.55 (0.46, 0.64)	0.37	0.77	
Sex	0.57 (0.50, 0.63)	0.29	0.85	
Smoking	0.53 (0.46, 0.60)	0.27	0.79	
Hypertension	0.51 (0.45, 0.58)	0.73	0.30	
Diabetes	0.52 (0.45, 0.59)	0.63	0.41	
Hypercholestrolemia	0.52 (0.45, 0.60)	0.42	0.63	
eGFR	0.54 (0.45, 0.63)	0.33	0.79	
LVEF ≤ 50%	0.53 (0.48, 0.57)	0.90	0.15	
Multivessel disease	0.55 (0.48, 0.63)	0.40	0.71	
FFR	0.83 (0.77, 0.90)	0.77	0.80	
CFR	0.87 (0.84, 0.91)	1.00	0.70	
IMR	0.83 (0.79, 0.88)	0.88	0.71	
Baseline coronary flow	0.64 (0.57, 0.71)	0.81	0.49	
Hyperemic coronary flow	0.90 (0.87, 0.93)	1.00	0.73	
Pre-PCI CFC	0.94 (0.93, 0.96)	1.00	0.88	

**D. ≥1 CFC category improvement**				

		**AUC (95% CI)**	**Sensitivity**	**Specificity**

Age		0.56 (0.51, 0.62)	0.58	0.55
Sex		0.51 (0.47, 0.54)	0.17	0.84
Smoking		0.51 (0.47, 0.55)	0.24	0.77
Hypertension		0.51 (0.47, 0.55)	0.71	0.30
Diabetes		0.54 (0.49, 0.58)	0.64	0.44
Hypercholestrolemia		0.56 (0.52, 0.61)	0.44	0.68
eGFR		0.52 (0.46, 0.57)	0.46	0.60
LVEF ≤ 50%		0.52 (0.48, 0.55)	0.17	0.87
Multivessel disease		0.54 (0.50, 0.59)	0.35	0.73
FFR		0.69 (0.64, 0.74)	0.56	0.76
CFR		0.81 (0.77, 0.85)	0.98	0.57
IMR		0.79 (0.75, 0.83)	0.81	0.66
Baseline coronary flow		0.58 (0.52, 0.63)	0.75	0.43
Hyperemic coronary flow		0.83 (0.80, 0.87)	1.00	0.53
Pre-PCI CFC		0.91 (0.88, 0.94)	1.00	0.79

***A,C,D**: Sensitivity and specificity are at the best cutoffs.*

***B:** R-squared was calculated for continuous changes in CFC categories, ranging –3 to +3.*

*CFC, coronary flow capacity; CFR, coronary flow reserve; EF, ejection fraction; FFR, fractional flow reserve; IMR, index of microvascular resistance; PCI, percutaneous coronary intervention.*

### Association Between Delta Coronary Flow Capacity and Incident Target Vessel Failure

**FIGURE 4:**
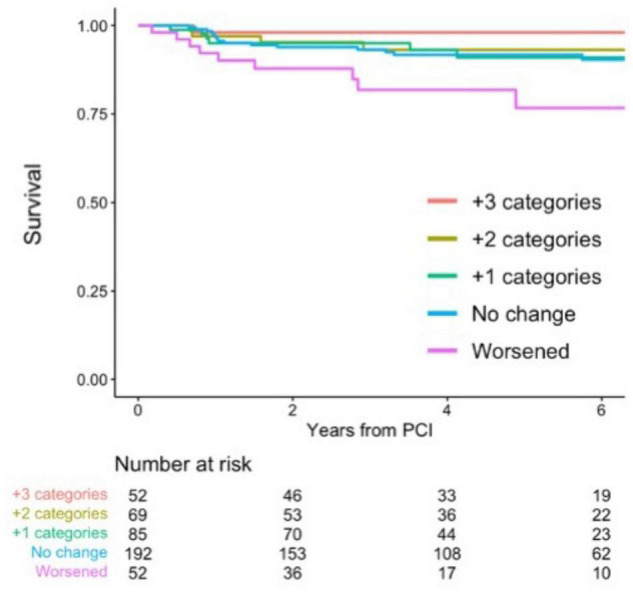
Survival from target-vessel failure (TVF) according to CFC changes. Kaplan–Meier curves showing survival from TVF according to the changes in CFC following PCI. CFC changes were categorized as worsened (-3 to -1 categories change), no change, +1, +2, or +3 categories improvement. Numbers indicate the number of patients at risk.

During a median follow-up of 4.3 (*IQR*: 2.5, 6.9) years, a total of 36 events were confirmed. Associations between CFC changes and detailed outcomes are summarized in Table 4. Patients with worsened, unchanged, +1, +2, and +3 improved CFC categories had the TVF risk of 17.3, 8.3, 7.1, 5.8, and 1.9%, respectively. Approximately 10% of the patients with worsened CFC had cardiac death or TVMI, whereas only one TVR event was observed in the 52 patients with +3 CFC categories improvement. Compared with no change in CFC categories after PCI, patients with category worsened, +1, +2, and +3 category improved had the hazard ratio (*HR*) (95% *CI*) for incident TVF of 2.27 (0.95, 5.43), 0.85 (0.33, 2.22), 0.45 (0.12, 1.63), and 0.14 (0.016, 1.30), respectively (*p* for linear trends = 0.0051; Table 5). After adjustment for confounders, one additional improvement in CFC status was associated with 0.61 (0.45, 0.83) times the hazard of TVF (*p* for linear trends = 0.0017). Figure 4 depicts the relevant Kaplan–Meier curves.

**TABLE 4:** Associations of CFC changes following PCI in ranks and detailed outcomes.

CFC change	Worsened	No change	+1 category improved	+2 category improved	+3 category improved
	*N* = 52	*N* = 192	*N* = 85	*N* = 69	*N* = 52
Target-vessel failure	9 (17.3)	16 (8.3)	6 (7.1)	4 (5.8)	1 (1.9)
Cardiac death	1 (1.9)	1 (0.5)	0 (0.0)	1 (1.4)	0 (0.0)
Target-vessel myocardial infarction	4 (7.7)	5 (2.6)	3 (3.5)	0 (0.0)	0 (0.0)
Target-vessel revascularization	4 (7.7)	10 (5.2)	3 (3.5)	3 (4.3)	1 (1.9)

*Values are n (%).*

*Target-vessel failure is a compostie of cardiac death, target-vessel myocardial infarction, and target-vessel revascularization.*

*CFC, coronary flow capacity; PCI, percutaneous coronary intervention.*

**TABLE 5:** Association between CFC changes following PCI and incident target vessel failure.

	A. Association between CFC changes and incident TVF						
CFC change	Continuous (per one category)	Worsened	No change	+1 category improved	+2 category improved	+3 category improved	*P*-trend
TVF case/*N*		9/52	16/192	6/85	4/69	1/52	
Unadjusted HR	0.67 (0.50, 0.88)	2.27 (0.95, 5.43)	ref	0.85 (0.33, 2.22)	0.45 (0.12, 1.63)	0.14 (0.016, 1.30)	0.0051
Multivariate-adjusted HR	0.61 (0.45, 0.83)						0.0017

**B. Coefficients in the multivariate regression models for CFC changes**							

				**HR**		**95% CI**	
Delta CFC, per category				0.62		0.47, 0.83	
Age, per year				1.00		0.97, 1.04	
Female				0.81		0.31, 2.15	
LAD versus RCA				0.74		0.34, 1.59	
LCx versus RCA				0.35		0.07, 1.61	
Diabetes				0.85		0.43, 1.67	
Multivessel disease				1.14		0.56, 2.32	
FFR, per 0.01 unit				0.97		0.94, 1.01	

*HRs were estimated using COX proportional hazard models categorical CFC changes with no change as the reference **(A)** and CFC improvement in ranks (ranging -3 to +3) **(A,B)**.*

*Multivariate models were adjusted for age (continuous), sex (male/female), diabetes (yes/no), vessel location (LAD/LCx/RCA), multivessel disease (yes/no), and FFR (continuous).*

*P-value for linear trend (P-trend) was calculated to estimate the statistical significance of the association between delta CFC (ranging -3 to +3) and incident TVF in the COX proportional hazard models.*

*CFC, coronary flow capacity; FFR, fractional flow reserve; HR, hazard ratio; LAD, left anterior descending artery; LCx, left circumflex artery; PCI, percutaneous coronary intervention; RCA, right coronary artery; TVF, target-vessel failure.*

**FIGURE 5:**
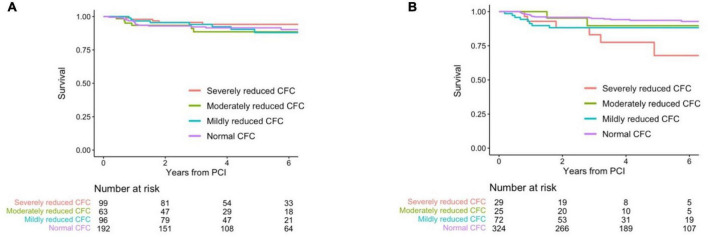
Kaplan–Meier curves showing survival from TVF according to the post-PCI CFC categories. Kaplan–Meier curves showing survival from TVF according to pre- **(A)** and post-CFC status **(B)**. CFC status was categorized as severely reduced, moderately reduced, mildly reduced, and normal. Numbers indicate the number of patients at risk.

No survival differences were observed according to distinct pre-PCI CFC status with a multivariable-adjusted *HR* of 1.25 (0.88, 1.77) for one rank higher CFC category (Tables 6A,B and Figure 5A). There were significant associations between post-PCI CFC and incident TVF (Tables 6C,D and Figure 5B).

**TABLE 6:** Association between pre- and post-PCI CFC and incident target vessel failure.

A. Association between pre-PCI CFC and incident TVF							
Pre-PCI CFC	Continuous (per one category)	Severely reduced	Moderately reduced	Mildly reduced	Normal	*P*-trend	
TVF case/*N*		5/99	6/63	8/96	17/192	
Unadjusted HR	1.14 (0.86, 1.52)	0.56 (0.20, 1.51)	1.15 (0.45, 2.93)	0.98 (0.42, 2.27)	ref	0.33
Multivariate-adjusted HR	1.25 (0.88, 1.77)					0.22

**B. Coefficients in the multivariate regression models for pre-PCI CFC**							

			**HR**		**95% CI**		

Pre-PCI CFC, per category			1.25		0.88, 1.77		
Age, per year			1.00		0.97, 1.04		
Female			0.85		0.32, 2.23		
LAD versus RCA			0.77		0.36, 1.67		
LCx versus RCA			0.31		0.07, 1.47		
Diabetes			0.96		0.49, 1.89		
Multivessel disease			1.09		0.54, 2.21		
FFR, per 0.01 unit			0.98		0.94, 1.03		

**C. Association between post-PCI CFC and incident TVF**							

**Post-PCI CFC**		**Continuous (per one category)**	**Severely reduced**	**Moderately reduced**	**Mildly reduced**	**Normal**	***P*-trend**

TVF case/*N*			6/29	2/25	8/72	20/324	
Unadjusted HR		0.66 (0.49, 0.88)	3.83 (1.54, 9.57)	1.38 (0.32, 5.89)	2.07 (0.91, 4.70)	ref	0.0050
Multivariate-adjusted HR		0.65 (0.48, 0.88)					0.0055

**D. Coefficients in the multivariate regression models for post-PCI CFC**							

			HR		95% CI	

Post-PCI CFC, per category			0.65		0.48, 0.88	
Age, per year			0.99		0.96, 1.03	
Female			0.79		0.30, 2.09	
LAD versus RCA			0.84		0.39, 1.81	
LCx versus RCA			0.40		0.09, 1.90	
Diabetes			0.80		0.40, 1.61	
Multivessel disease			1.08		0.53, 2.20	
FFR, per 0.01 unit			1.00		0.97, 1.04	

*HRs were estimated using COX proportional hazard models for categorical pre-PCI CFC with normal CFC as the reference **(A)**, continuous pre-PCI CFC in ranks (ranging 1–4) (**A,B**), categorical post-PCI CFC with normal CFC as the reference **(C)**, continuous post-PCI CFC in ranks (ranging 1–4) **(C,D)**.*

*Multivariate models were adjusted for age (continuous), sex (male/female), diabetes (yes/no), vessel location (LAD/LCx/RCA), multivessel disease (yes/no), and FFR (continuous).*

*P-value for linear trend was calculated to estimate the statistical significance of the association between CFC in ranks (ranging 1–4) and incident TVF in the COX proportional hazard models.*

*CFC, coronary flow capacity; FFR, fractional flow reserve; HR, hazard ratio; LAD, left anterior descending artery; LCx, left circumflex artery; PCI, percutaneous coronary intervention; RCA, right coronary artery; TVF, target-vessel failure.*

Table 7 shows the prediction of incident TVF by various nested models and the metrics for the improvement in the discrimination. Models comprised solely of demographics had *AUC* (95% *CI*) of 0.57 (0.47, 0.67) and the further consideration of pre-PCI FFR and pre-PCI CFR did not improve the discrimination. The model additionally including delta CFC had higher *AUC* (0.71 [95% *CI*: 0.62, 0.79]) and the discrimination was well improved compared with the model without delta CFC (*NRI*: 0.47 [95% *CI*: 0.14, 0.81] and *IDI*: 0.035 [95% *CI*: 0.011, 0.060]).

**TABLE 7:** Prediction incident target vessel failure based on pre-PCI information.

	AUC (95% CI)	Comparator of NRI/IDI analyses	Continuous NRI (95% CI)	IDI (95% CI)
Model 1: Demographics	0.57 (0.47, 0.67)	–	–	–
Model 2: Model 1 + pre-PCI FFR	0.57 (0.47, 0.67)	Model 1	0.00 (–0.33, 0.33)	0.000 (0.000, 0.000)
Model 3: Model 2 + pre-PCI CFR	0.59 (0.50, 0.68)	Model 2	–0.01 (–0.35, 0.33)	0.002 (–0.002, 0.006)
Model 4: Model 3 + delta CFC	0.71 (0.62, 0.79)	Model 3	0.47 (0.14, 0.81)	0.035 (0.011, 0.060)

*Models were based on logistic regressions for incident target vessel failure.*

*Model 1 included age, sex, diabetes, vessel location, and multivessel disease; Model 2 was Model 1 plus pre-PCI FFR; Model 3 was Model 2 plus pre-PCI CFR (continuous); and Model 4 was Model 3 plus delta CFC (in ranks, ranging –3 to +3).*

*AUC, area under the curve; CFC, coronary flow capacity; CFR, coronary flow reserve; IDI, integrated discrimination improvement; NRI, net reclassification improvement; PCI, percutaneous coronary intervention.*

## Discussion

**FIGURE 6:**
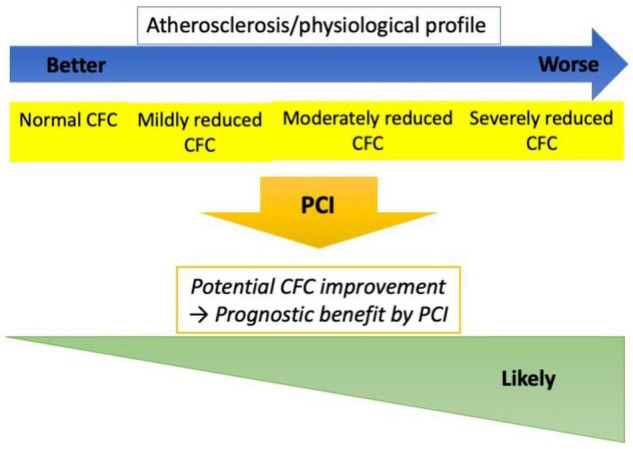
Study summary. Worse CFC status was associated with higher atherosclerotic risk and worse coronary physiological profile. However, the clinical courses were similar across the pre-PCI CFC if treated by elective PCI, while the changes in CFC following PCI were robustly associated with the risk of TVF. Furthermore, CFC changes are largely determined by the pre-PCI status. These observations implicate that lesions with reduced CFC could potentially attain greater CFC improvement by PCI, and consequently the lowered risk of vessel-oriented adverse events. CFC could thus serve as guidance for elective PCI indication, which needs to be evaluated in studies comparing PCI versus medical therapy in reduced CFC lesions.

In the present study, the changes in CFC status following PCI were robustly associated with incident TVF in patients with the CCS. The change was largely determined by the pre-PCI CFC status. Furthermore, no association between pre-PCI CFC and incident TVF was observed, suggesting prognostic benefits of PCI in patients with reduced CFC categories. This study provides a mechanistic explanation on potential favorable effects of PCI on reducing vessel-oriented outcomes in lesions with reduced CFC, supporting a use of CFC, in addition to FFR, in guiding PCI to maximize the benefit. A summary of the present study was illustrated in Figure 6.

Although FFR well captures the severity of epicardial atherosclerosis, the index does not directly incorporate the information on coronary flow and microvascular resistance. CFR has been attracted as a potential flow-related marker that could guide PCI, while a recent prospective study did not observe the role (18). This is partly because low CFR is a heterogeneous condition with varied resting and hyperemic coronary flow status (18). The physiological benefit of PCI primarily lies in modifying the hyperemic flow limitation (19–21). CFC is an integrated concept of CFR and hyperemic coronary flow, and thus low FFR combined with reduced CFC highlights hyperemic coronary flow limitation-based ischemia due to epicardial atherosclerosis, where PCI could maximally offer the physiological benefit. In accordance with the theoretical basis, we have previously showed a differential prognostic effect of PCI according to the CFC status in registry data (13). The present study further supports the role of CFC by highlighting the impact of changes in CFC status following PCI, a direct representation of the improvement in coronary flow and ischemic burden; notedly, TVF was observed in only 1 out of 52 patients whose CFC status changed from severely reduced to normal. The pre-PCI CFC status largely offers the prediction of the changes, supportive of the usage in guiding PCI.

From another aspect, current FFR-guidance might indicate too many stable vessels for revascularization in which the physiological benefit from PCI could not be expected. In particular, lesions with low FFR and normal CFC, comprising 43% of vessels in the present registry, hardly anticipates coronary flow restoration or reduction of ischemic burden, and thus these might better be treated medically with respect to prognostic advantage and possibly to symptomatic relief. Although there is a correlation between FFR and CFC, FFR only explains 12% of the variability of CFC changes, supportive of the merit of integrating CFC for guiding PCI indication in addition to FFR. Additionally, while this study demonstrated a clear prognostic contribution of the changes in the regional CFC following PCI, the impact of PCI on the global coronary flow property could be different as we previously described (19, 22, 23). Further consideration of global physiological indices might lead to better identification of patients with CCS who would likely benefit from the intervention.

The study has several limitations. The present analysis is based on a single-center registry and as such the generalizability is limited. The moderate sample size prevents rigorous adjustments of confounders. However, such adjustments could make the estimate further away from null, as higher CFC improvement can occur in patients with pre-PCI reduced CFC, which categories were generally associated with higher atherosclerotic risks and worse physiological profiles. Thermodilution methods could overestimate CFR compared with Doppler-technique (24). Finally, the present study does not directly indicate the usefulness of CFC in guiding PCI but offers an explanation on the potential mechanisms, i.e., improvement in CFC status. Another study is needed to demonstrate the prognostic impact of FFR plus CFC-guided compared with FFR only-guided PCI in a larger population.

## Conclusion

Changes in CFC categories following PCI was associated with lower risk of incident TVF in patients with CCS who underwent PCI. The pre-PCI CFC status was a sole strong predictor for the CFC changes. This study provides a mechanistic explanation on a potential favorable effect of PCI on reducing vessel-oriented outcome in lesions with reduced CFC and low FFR.

## Data Availability Statement

The raw data supporting the conclusions of this article will be made available by the authors, without undue reservation.

## Ethics Statement

The studies involving human participants were reviewed and approved by Tsuchiura Kyodo General Hospital Ethics Committee. The patients/participants provided their written informed consent to participate in this study.

## Author Contributions

RH: concept, design, drafting of the manuscript, and statistical analysis. All authors: acquisition, analysis, or interpretation of data. TK: critical revision of the manuscript for important intellectual content, administrative, technical, or material support, and supervision. All authors contributed to the article and approved the submitted version.

## Conflict of Interest

The authors declare that the research was conducted in the absence of any commercial or financial relationships that could be construed as a potential conflict of interest.

## Publisher’s Note

All claims expressed in this article are solely those of the authors and do not necessarily represent those of their affiliated organizations, or those of the publisher, the editors and the reviewers. Any product that may be evaluated in this article, or claim that may be made by its manufacturer, is not guaranteed or endorsed by the publisher.

## Abbreviations

bCF: basal coronary flow
bTmn: basal mean transit time
CFC: coronary flow capacity
CFR: coronary flow reserve
FFR: fractional flow reserve
hCF: hyperemic coronary flow
hTmn: hyperemic mean transit time
PCI: percutaneous coronary intervention
TVF: target vessel failure.
